# Serum TGF-β1 as a Biomarker for Type 2 Diabetic Nephropathy: A Meta-Analysis of Randomized Controlled Trials

**DOI:** 10.1371/journal.pone.0149513

**Published:** 2016-02-22

**Authors:** Xin Mou, Di-Yi Zhou, Dan-Yang Zhou, Jing-Ru Ma, Ying-Hui Liu, Hui-Ping Chen, Yong-Bin Hu, Cheng-Min Shou, Jia-Wei Chen, Wen-Hong Liu, Guo-Ling Ma

**Affiliations:** 1 Endocrinology Department, Hangzhou Red Cross Hospital, Hangzhou, 310003, China; 2 Zhejiang Chinese Medical University, City Road 548, Binjiang District, Hangzhou City, Zhejiang Province, 310053, China; Mario Negri Institute for Pharmacological Research and Azienda Ospedaliera Ospedali Riuniti di Bergamo, ITALY

## Abstract

**Background:**

Abnormal expression of serum TGF-β1 was found in patients with diabetic nephropathy. However, the association of TGF-β1 with the risk of diabetic nephropathy remains unknown. The present study was undertaken to investigate whether such an association exists.

**Methods:**

We searched the Chinese VIP, Wangfang, China National Knowledge Infrastructure, PubMed, Embase, and Google Scholar databases for relevant studies and extracted all eligible data. Stata12 software was used for statistical analysis.

**Results:**

Nine reports met our criteria and were used for data extraction. There were 264 patients and 227 healthy controls from qualified reports in this meta-analysis. The results suggested that serum TGF-β1 levels were significantly up-regulated in patients with diabetic nephropathy; the instrumental variable was 3.94 (95% confidence interval 3.20–4.68, p<0.01).

**Conclusions:**

Meta-analysis suggested that elevated serum TGF-β level in patients with diabetes is associated with a high risk of nephropathy. Further studies are required to validate these observations.

## Introduction

Diabetic nephropathy, one of leading causes of death in patients with diabetes, is a progressive kidney disease caused by damage to the capillaries in the kidneys’ glomeruli [1]. The prevalence of diabetic nephropathy is rising in developed countries, and it was reported to be the primary cause for end-stage renal disease in diabetic patients worldwide [1, 2]. Moreover, the high incidence of diabetic nephropathy, its poor prognosis, and its high cost of treatment has caused it to become a public health issue. Therefore, early diagnosis of diabetic nephropathy is required to promptly intervene and prevent or delay deterioration due to the disease [3]. Currently, microalbuminuria is a widely-used early marker for nephropathy in diabetic patients [3]. However, the sensitivity and accuracy of microalbuminuria as a predictor has been questioned in recent years [3, 4]. Therefore, novel biomarkers with the ability to predict disease progression accurately are needed in clinic practice.

The glomerular capillary wall consists of podocytes (or visceral epithelial cells), glomerular endothelial cells, glomerular basement membranes, and mesangial cells [5]. One of the main characteristics of diabetic nephropathy is the expansion of the mesangial matrix which leads to the subsequent accumulation of mesangial cell-derived extracellular matrix (ECM) components [6]. During this process, members of the transforming growth factor-β (TGF-β) family are thought to play an indispensable role [7]. Generally, TGF-β family proteins are essential for regulating cellular growth, differentiation, autophagy, and apoptosis, as well as immune suppression [8, 9]. However, TGF-β1 has also been recognized as a key mediator in ECM formation [10]. Up-regulation of TGF-β1 expression is reported to be indispensable in fibrosis and in tissue remodeling in various organs during disease progression [11], including glomerular fibrosis in the kidney [12].

Currently, evidence from various studies have implicated TGF-β1, as well as its associated signaling pathways, were associated with diabetic nephropathy [13–16]. Moreover, there are also reports of abnormal expression of TGF-β1 in the serum and urine of patients with diabetic nephropathy [17–19]. However, due to the limited clinical samples in individual studies, it is difficult to reach consensus regarding the relationship between serum TGF-β1 level and diabetic nephropathy. Therefore, we conducted a meta-analysis based on a combination of a number of relevant reports to determine if serum TGF-β1 could serve as a novel biomarker for testing for the early occurrence of diabetic nephropathy.

## Methods

### Literature searching

A literature search was conducted using the Chinese VIP, Wangfang, China National Knowledge Infrastructure, PubMed, Google Scholar, and Embase databases for randomized controlled trials by using the keywords “Transforming Growth Factor” and “Diabetic Nephropathy” through June 2015. There were no language preferences.

### Inclusion and exclusion criteria

Eligible studies included in this meta-analysis met the following criteria: (1) patients with diabetic nephropathy were enrolled; (2) the study included at least two case control groups: healthy control subjects and patients with type 2 diabetes but without nephropathy; and (3) serum TGF-β1 levels (mean ± SEM) were reported in the study. Exclusion criteria included: (1) reviews, editorials, conference reports, and dissertations; 2) studies conducted in animal models (mice, rats, rabbits, and others); 3) cell line-based or in-vitro studies; and 4) duplicate publications (i.e., in multiple languages).

### Data extraction

Two reviewers independently extracted the data using a special data extraction form. The following information was recorded: (1) first author’s family name and publication year; 2) average age and ethnicity of patients; (3) sample size of each group; (4) diagnosis markers and discrete value of serum TGF-β1 levels. Disagreements were resolved by the intervention of a third reviewer.

### Outcomes and statistical analysis

The main outcome was the comparison of serum TGF-β1 levels in diabetic nephropathy patients to those in healthy controls and type 2 diabetes patients without nephropathy. Random modeling was performed by using Stata software version 12.0 (StataCorp LP, College Station, Texas) for meta-analysis. Weighed mean differences, standardized mean differences, and 95% confidence intervals (CI) for the outcome of continuous variables were determined. The value of I^2^ was assessed for heterogeneity; if the I^2^ value was greater than 50%, efforts were made to determine the source of heterogeneity by sensitivity or subgroup analysis to identify outliers. The S1 PRISMA Checklist (2009) was list at the end of this paper.

## Results

### Study selection and characteristics

**Fig 1:**
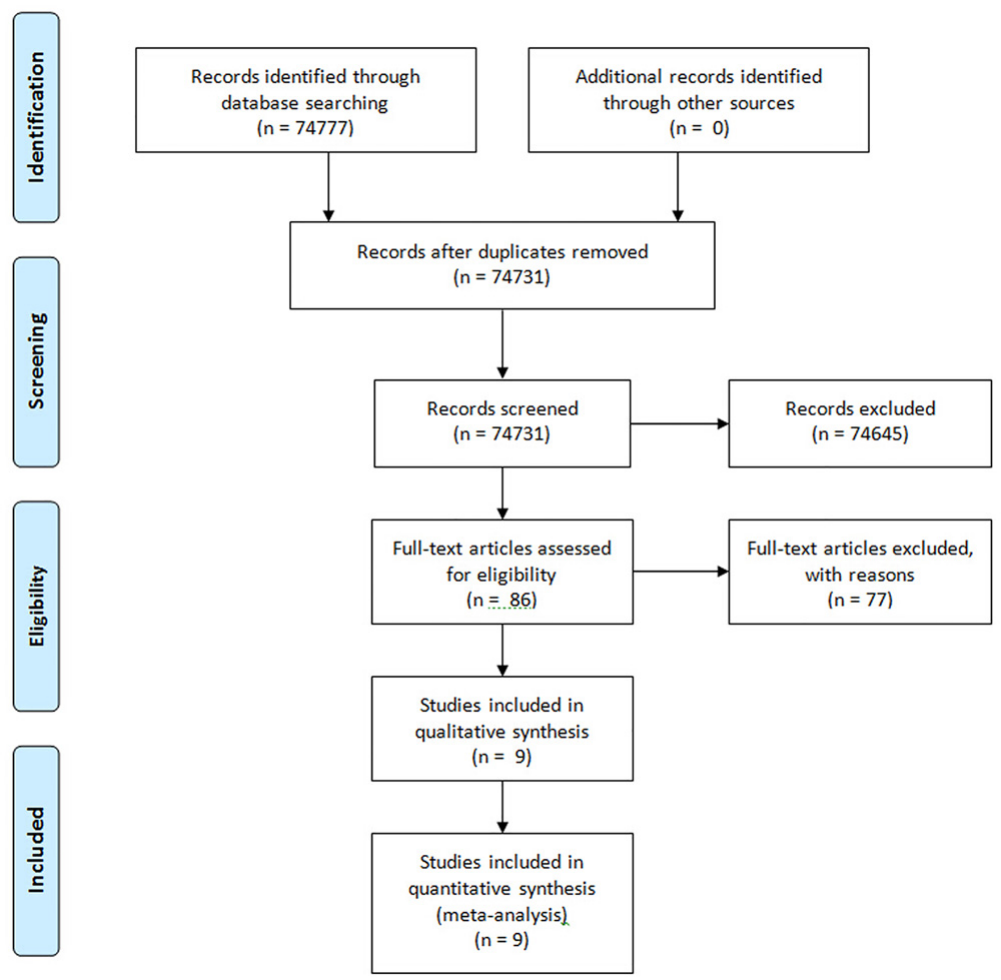
Search flow diagram for retrieving relevant studies.

As shown in the flow diagram in Fig 1, a total of 74777 articles were obtained from the initial database search. After perusing the title and abstracts, 132 randomized controlled trials deemed relevant for TGF-β1 level analysis in diabetic nephropathy patients remained for additional reviewing. After excluding duplicates, 86 articles were fully read. Of these, nine met the inclusion criterion, all of which involved studies conducted in China.

Data from the included articles were extracted and are shown in Table 1. The average ages of the patients tended to be similar. Furthermore, some studies (including Ju 2001, Li 2011, Zhang 2004, and Zhang 2008) divided diabetic nephropathy patients into two groups: a microalbuminuria group (20 μg/min < UREA < 200 μg/min) and a clinical diabetic nephropathy group (UREA >200 μg/min) depending on urine microalbumin levels as measured every 24 h.

**Table 1:** Characteristics of included studies.

Study	Year	Ethnicity	Healthy subjects			Type 2 diabetes				
			Total (M/F)	Average Age	Source of control	Total (M/F)	Average Age	DM	DN	Duration (Years)
Fu et al.[20]	2007	Asian	35 (24/11)	50.2	hospital	65 (42/23)	55.4	34	31	10.6
Ju et al.[18]	2001	Asian	15 (7/8)	53.1	hospital	45 (21/24)	56.9	14	31	9.9
Li et al.[21]	2011	Asian	31 (19/12)	56.2	hospital	98 (51/47)	57.5	32	66	2–20
Liu et al.[22]	2011	Asian	20 (10/10)	55	hospital	95 (50/45)	56	32	63	2~20
Wang et al.[23]	2008	Asian	35	UD	hospital	76	UD	44	32	UD
Wu et al.[19]	2011	Asian	35 (25/10)	65.5	hospital	102 (52.50)	66.4	69	33	11.2
Xie et al.[24]	2006	Asian	30 (18/12)	50	hospital	105 (58/47)	58	60	45	12
Zhang et al.[17]	2004	Asian	16 (8/8)	UD	hospital	76 (37/39)	UD	25	51	1–20
Zhang et al.[25]	2008	Asian	26	UD	hospital	100 (52/48)	58.5	30	70	UD

M: Male, F: Female, DM: Diabetic mellitus, DN: diabetic nephropathy, UD: undisclosed

### Association of serum TGF-β1 levels with the risk diabetic nephropathy

**Fig 2:**
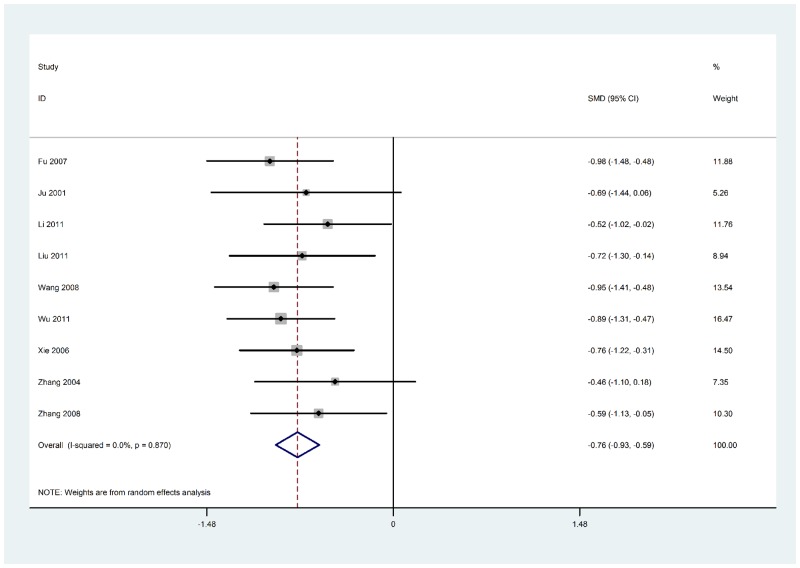
Meta-analysis of the association of serum TGF-β1 levels with the risk of diabetic nephropathy. SMD, standardized mean difference.

A total of 264 patients with diabetic nephropathy and 227 healthy controls were included in this meta-analysis. As shown in Fig 2, the instrumental variable (IV) was 3.94, (95% CI 3.20–4.68, P<0.01) which indicated that serum TGF-β1 levels were significantly increased in patients with diabetic nephropathy. The total I^2^ value was 83.6% (P<0.01), suggesting a significant heterogeneity in all the included studies. Moreover, the instrumental variable (IV) was -0.76, (95% CI -0.93 to -0.59, P>0.5) indicates that that serum TGF-β1 levels were not changed in patients with only Type II diabetic.

### Subgroup analysis for the heterogeneity of included studies

**Fig 3:**
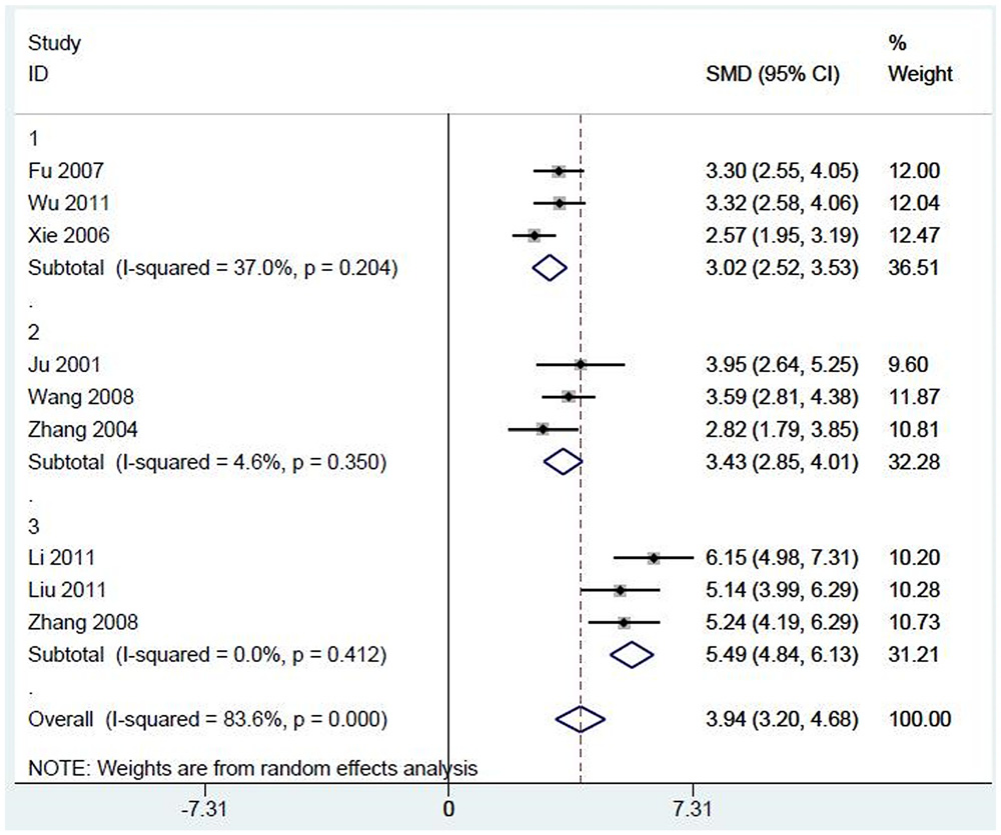
Meta-analysis of the association of serum TGF-β1 levels with the risk of diabetic nephropathy: subgroup analysis according to diagnostic criteria. SMD, standardized mean difference.

We next strove to identify the source of heterogeneity. In the studies by Wu et al., Fu et al., and Xie et al., no detailed information was provided regarding the criteria for diagnosing diabetic nephropathy. In the studies by Ju et al., Wang et al., and Zhang et al. (2004), the average urinary protein in diabetic nephropathy patients was above 300 mg per 24 h. However, for the other studies, the urinary protein in the experimental groups ranged between 30 and 300 mg per 24 h. Therefore, we divided diabetic nephropathy patients from these studies into three subgroups depending on the urinary protein quantity. As shown in Fig 3, the SMD was 3.02 (95% CI 2.52–3.53, p<0.01) and the I^2^ = 37% in group 1; the SMD was 3.43 (95% CI 2.85–4.01, p<0.01) and the I^2^ = 4.6% in group 2; and the SMD was 5.49 (95% CI 3.20–4.68, p<0.01) and I^2^ = 0% in group 3. These results indicated that the criteria used for diagnosing diabetic nephropathy may be responsible for the high heterogeneity in these studies.

### Evaluation of publication bias

As positive results are more readily to be accepted and negative results may be omitted, accounting for the possible publication bias in our included study, therefore, publication bias was tested by Begg’s funnel plots and Egger’s test. No obvious publication bias was observed for the overall population (Begg’s p = 0.048 and Egger p = 0.019) which indicate that the conclusion we draw from our results believable.

## Discussion

The activation of the TGF-β1 signaling pathway in the renal system was found to be an intermediary step in diabetic kidney injury. TGF-β1 not only stimulates the synthesis of some key components of the ECM, such as type I and type IV collagen, but also decreases matrix degradation by inhibiting the protease activity [26, 27]. Many studies have shown that the outcome of diabetic nephropathy is often linked to the abnormal expression of TGF-β1 in both animal models and human subjects. Moreover, long-term administration of anti-TGF-β1 antibody to block the TGF-β1 mediated signaling pathway in animal models prevented mesangial matrix expansion [28, 29]. However, due to the limited samples sizes in individual publications, it remains inconclusive whether up-regulated serum TGF-β1 is associated with a high risk of diabetic nephropathy.

By applying meta-analysis, a powerful tool which can combine the results of different randomized controlled studies, nine reports with 264 cases and 227 healthy controls were analyzed to investigate whether elevated serum TGF-β1 is a risk factor for the development of diabetic nephropathy. Our results showed that serum TGF-β1 was significantly up-regulated in patients with diabetic nephropathy (p<0.01), and that serum TGF-β1 does represent a risk factor for the occurrence of diabetic nephropathy. As the population sample of this meta-analysis was sufficiently large (>200), the results of our meta-analysis are persuasive. Moreover, no significant publication bias was observed when tested using Begg’s funnel plots and Egger’s test.

High heterogeneity was found when analyzing all the included studies; the I^2^ value was 83.6%. By analyzing the data in detail, we concluded that the heterogeneity was due to the different criteria used to diagnose diabetic nephropathy, as well as the fact that data was extracted from patients with different stages of nephropathy. Some studies had not adequately described how diabetic nephropathy was diagnosed and lacked discrete urinary protein values, while patients in other studies were divided into different stages of nephropathy depending on their urinary microalbuminuria gradient. The I^2^ values in the three subgroups (the third being studies not included in the two aforementioned groups) did not exceed 50%.

**Fig 4:**
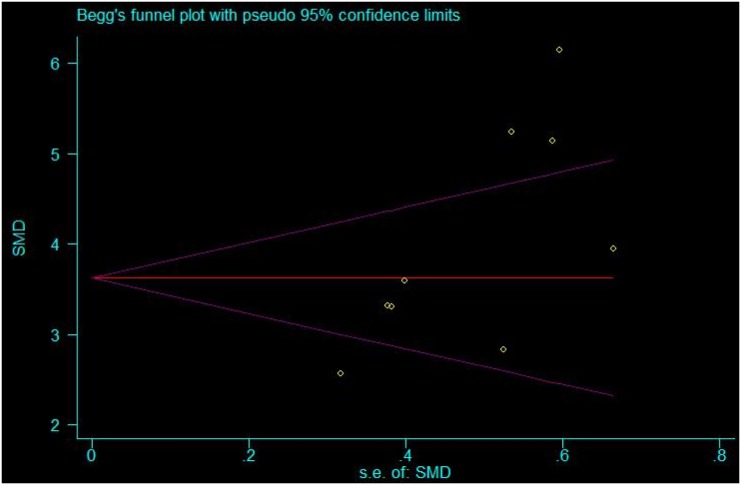
Begg’s funnel plot for evaluating publication bias in the included studies.

Previous meta-analyses had reported the association of the *TGF-β1 T869C* gene polymorphism with diabetic nephropathy risk. A survey of eligible studies from PubMed, the Cochrane Library, and the Chinese Biological Medicine Database found the TT genotype of *TGF-β1 T869C* was associated with diabetic nephropathy risk in the overall population. This suggested a potential role for TGF-β1 gene expression in the progression of diabetic nephropathy [30]. Another study also provided a similar conclusion [31]. Concordantly, our results highlighted the correlation of TGF-β1 protein levels with the risk of diabetic nephropathy. However, certain limitations should be considered in regards to this study. Although the p value (<0.01) from the Begg’s funnel plots and Egger’s test suggested no publication bias, we still noticed asymmetry in the funnel plot graph (Fig 4). Therefore, other relevant published or unpublished studies with null results may have been omitted.

In conclusion, our study is the first meta-analysis to have assessed the association between serum TGF-β1 levels and diabetic nephropathy risk. The results suggest that up-regulation of serum TGF-β1 is associated with an increased risk of diabetic nephropathy. Larger-scale case controlled studies are required to confirm these findings.

## Supporting Information

S1 PRISMA Checklist PRISMA 2009 Checklist. (DOC)
